# Prevalence of asymptomatic celiac disease in children with fibromyalgia: a pilot study

**DOI:** 10.1186/1546-0096-9-11

**Published:** 2011-06-13

**Authors:** Bruce Taubman, Peter Mamula, David D Sherry

**Affiliations:** 1 Division of General Pediatrics, University of Pennsylvania School of Medicine, The Children's Hospital of Philadelphia, Advocare Cherry Hill Pediatric Group, Cherry Hill, NJ, USA; 2 Division of Gastroenterology and Nutrition, University of Pennsylvania School of Medicine, The Children's Hospital of Philadelphia, Philadelphia, PA, USA; 3 Division of Rheumatology, University of Pennsylvania School of Medicine, The Children's Hospital of Philadelphia, Philadelphia, PA, USA

## Abstract

**Background:**

The objective of this study was to prospectively determine the prevalence of asymptomatic celiac disease among children presenting with fibromyalgia. The secondary objective was to investigate if their symptoms resolved on a gluten free diet.

**Findings:**

All children seen in the Amplified Musculoskeletal Pain clinic between the ages of 12 and 17 years of age who fulfilled the 1990 American College of Rheumatology diagnostic criteria for fibromyalgia were invited to participate. A total immunoglobulin A (IgA) level, IgA antiendomysial (EMA) and IgA anti-TTG antibodies was obtained on all study subjects. A visual analog scale for pain and a functional disability inventory were obtained on all patients. If a patient had elevated EMA or TTG a small bowel biopsy was done. Patients with celiac disease were placed on a gluten-free diet and observed to see if their symptoms resolved.

50 patients, 45 females, completed the study. Only one patient was found to have celiac disease. On a gluten-free diet her tissue transglutaminase antibody level returned to normal but her visual analog scale scores increased and her functional disability inventory was 40 initially and 21 at follow up.

**Conclusions:**

In this pilot, single center study at a tertiary children's hospital patients with fibromyalgia do not seem to have occult celiac disease at an increased rate over the population as a whole.

## Introduction

One of the forms of diffuse amplified musculoskeletal pain of unknown etiology, fibromyalgia, occurs in both children and adults. Zipser et al, by internet questionnaire, found that among 134 adults with celiac disease the initial physician diagnosis in 9% of patients was fibromyalgia [1]. Bonakdar lists celiac disease as a predisposing condition for fibromyalgia and West notes that celiac disease through the mechanism of vitamin D deficiency can cause symptoms mimicking fibromyalgia [2,3]. The internet is replete with references that fibromyalgia may be undiagnosed celiac disease. We have seen one child with celiac disease who while consuming spelt, believing it to be gluten free, developed markedly elevated anti-tissue transglutaminase antibody (TTG) levels and symptoms of widespread body pain consistent with fibromyalgia. Her TTG level returned to normal when spelt was removed from her diet and the musculoskeletal symptoms resolved. Therefore, we sought to investigate if there is an increase in occult celiac disease in children with fibromyalgia. The primary aim of the study was to determine the prevalence of children with asymptomatic celiac disease among patients presenting with fibromyalgia and the secondary outcome was to investigate if their symptoms resolve on a gluten-free diet,

## Patients and methods

All children seen in the Amplified Musculoskeletal Pain clinic between the ages of 12 and 17 years of age who fulfilled the diagnostic criteria for fibromyalgia were invited to participate by one of the authors (DDS). The American College of Rheumatology criteria was used to define fibromyalgia; generalized musculoskeletal pain over at least half their body and pain at a minimum of 11 of 18 pressure points (applying 3-4 Kg). Digital pressure or less) without other sources of the pain for at least 3 months [4]. These patients had quite typical fibromyalgia and many complained of sleep, thinking, and multiple somatic problems. Patients were excluded from the study if they had previously been tested for celiac disease, had celiac disease, or were on a gluten-free diet. Informed consent was obtained from the parents and assent was obtained from the patient. Patients were reimbursed $10 for their time. The institutional review board of The Children's Hospital of Philadelphia approved this study.

A celiac panel consisting of total immunoglobulin A (IgA) level, IgA antiendomysial (EMA) and IgA anti-TTG antibodies was obtained on all study subjects. If the patient was IgA deficient a blood test for IgG EMA and TTG antibodies was performed [5]. As per our standard of care a visual analog scale (VAS) for pain (10 being the worse) and a functional disability inventory (FDI) (60 being the most disabled) were obtained on all patients [6,7]. If a patient had elevated EMA or TTG a small bowel biopsy was done to confirm the diagnosis of celiac disease [5]. Patients with confirmed celiac disease were placed on a gluten-free diet and EMA and or TTG antibodies levels were obtained every 3 months. Once the patient was free of GI symptoms and had normal celiac antibody levels his/her celiac disease was considered in good control. At that time VAS and FDI scores were obtained,. The study design is a single site cross-sectional serologic survey. We assumed the hypothesis that celiac disease can present as fibromyalgia to be true. It was also assumed that this association is to the degree that 9% of patients presenting with fibromyalgia will have celiac disease. The true prevalence of celiac disease in the United States is unknown. After reviewing the various estimates in the literature we chose to use the value of 0.9%for this study [8,9].The sample size using these parameters which can give 80% statistical power while controlling onesided, = 0.05, is 47.

## Results

Over a 4 year period 54 patients were enrolled in the study and 4 patients withdrew. Two patients dropped out because of fear of venipuncture and 2 after the first attempt to draw blood failed. Among the 50 patients who completed the study 46 were female and the mean age was 14.9 years, with a range 3-17 years. The average pain duration was 20 months with a range of 5 to 143 months. As is typical for our population, 47 were Caucasian and the remainder were African American. No patient was IgA deficient. Only one patient had a positive celiac serology with an anti-TTG of 219 and an anti-EMA of 1:320. Celiac disease was confirmed by small bowel biopsy. On a gluten-free diet her TTG antibodies returned to normal but her pain VAS scores increased from 7out of 10 to 10 out of 10 and her functional disability inventory was 40 initially and 21 at follow up.

## Discussion

This pilot study failed to demonstrate that in 50 children with fibromyalgia, there was an increase in occult celiac disease. The possibility that this occurs at a much smaller rate then 9% as the study design assumed cannot be ruled out. To determine this a much larger study population would be necessary. Another possibility not explored by this study is that celiac disease causes musculoskeletal symptoms but not enough to fit the definition of fibromyalgia. Adults with celiac disease would have had a much longer underlying, untreated gastrointestinal disease and therefore the musculoskeletal manifestations may differ from children who present earlier. Our single patient identified with celiac, has never had symptoms or signs suggestive of celiac disease.

## Conclusion

In this pilot study at a tertiary children's hospital patients with fibromyalgia do not seem to have occult celiac disease at an increased rate over the population as a whole.

## Abbreviations

AS: Visual Anolog Scale; EMG: Anti-endomysial antibody; FDI: Functional Disability Inventory; IgA: Immunoglobulin A; TTG: Anti-tissue transglutaminase antibody.

## Competing interests

The authors declare that they have no competing interests.

## Authors' contributions

BT contributed to conception and design of the study, interpretation of data and was involved in drafting the manuscript and revising it critically for important intellectual content; and gave final approval of the version to be published. PM contributed to conception and design of the study, interpretation of data and was involved in drafting the manuscript and revising it critically for important intellectual content; and gave final approval of the version to be published. DDS contributed to conception and design of the study, collection and interpretation of data and was involved in drafting the manuscript and revising it critically for important intellectual content; and have gave final approval of the version to be published.
